# Molecular Hydrogen as a Medical Gas for the Treatment of Myalgic Encephalomyelitis/Chronic Fatigue Syndrome: Possible Efficacy Based on a Literature Review

**DOI:** 10.3389/fneur.2022.841310

**Published:** 2022-04-11

**Authors:** Shin-ichi Hirano, Yusuke Ichikawa, Bunpei Sato, Yoshiyasu Takefuji, Fumitake Satoh

**Affiliations:** ^1^Department of Research and Development, MiZ Company Limited, Kamakura, Japan; ^2^MiZ Inc., Newark, CA, United States; ^3^Professor Emeritus, Keio University, Tokyo, Japan; ^4^Faculty of Data Science, Musashino University, Tokyo, Japan

**Keywords:** molecular hydrogen, myalgic encephalomyelitis (ME), chronic fatigue syndrome (CFS), hydroxyl radicals, mitochondrial dysfunction, oxidative stress, post COVID, long COVID

## Abstract

Myalgic encephalomyelitis/chronic fatigue syndrome (ME/CFS) is a disorder that is characterized by fatigue that persists for more than 6 months, weakness, sleep disturbances, and cognitive dysfunction. There are multiple possible etiologies for ME/CFS, among which mitochondrial dysfunction plays a major role in abnormal energy metabolism. The potential of many substances for the treatment of ME/CFS has been examined; however, satisfactory outcomes have not yet been achieved. The development of new substances for curative, not symptomatic, treatments is desired. Molecular hydrogen (H_2_) ameliorates mitochondrial dysfunction by scavenging hydroxyl radicals, the most potent oxidant among reactive oxygen species. Animal experiments and clinical trials reported that H_2_ exerted ameliorative effects on acute and chronic fatigue. Therefore, we conducted a literature review on the mechanism by which H_2_ improves acute and chronic fatigue in animals and healthy people and showed that the attenuation of mitochondrial dysfunction by H_2_ may be involved in the ameliorative effects. Although further clinical trials are needed to determine the efficacy and mechanism of H_2_ gas in ME/CFS, our literature review suggested that H_2_ gas may be an effective medical gas for the treatment of ME/CFS.

## Introduction

The core symptoms of myalgic encephalomyelitis/chronic fatigue syndrome (ME/CFS) are severe fatigue that persists for more than 6 months, extreme exhaustion after exertion, memory impairment, difficulty concentrating, and sleep disturbances (1, 2). It is also an intractable disease that is sometimes accompanied by headache, arthralgia, myalgia, gastrointestinal symptoms, immune system abnormalities, and hypersensitivity to light, sound, smell, and chemicals (1, 2). Symptoms vary in appearance and severity from patient to patient, but decrease overall quality of life as well as social, occupational, and personal activities, and some patients may even become bedridden (1, 2). Although the objective abnormality of ME/CFS has been questioned, recent developments in neuroimaging as well as analytical techniques for blood markers, energy metabolism, and mitochondrial functions have provided evidence for biological abnormalities in this disease (3, 4). Many studies have implicated disorders in the structure and functions of mitochondria, which are responsible for abnormal energy metabolism, in the pathogenesis of ME/CFS (5–25). The prevalence of ME/CFS is estimated to be 0.1–0.5% of the population (26, 27). The number of patients in the United States (US) is estimated to be between 836,000 and 2.5 million (4). The direct and indirect economic cost of ME/CFS in the US has been reported to be as high as 17–24 billion US dollars per year (4).

Molecular hydrogen (H_2_) is a flammable, colorless, odorless, tasteless, and non-toxic gaseous molecule. It functions as an antioxidant that selectively scavenges reactive oxygen species (ROS) and reactive nitrogen species with very high oxidative capacity, namely, hydroxyl radicals (·OH) and peroxynitrite, respectively (28). H_2_ has been shown to exert therapeutic effects on various diseases, such as cancer (29–32), cardiovascular disease (33, 34), neurological disease (35, 36), respiratory disease (37, 38), and metabolic syndrome (39, 40). The effects of H_2_ are not limited to antioxidant activity, they also include anti-inflammatory, anti-apoptotic, and anti-allergic activities, improvements in lipid metabolism, and the regulation of gene expression and signal transduction (41, 42). In mammalian cells, H_2_ is an inactive molecule that has no metabolic system and does not react with biological substances; however, it reacts with ·OH, which is abundant in mitochondria (43). H_2_ easily passes through the blood-brain barrier. Due to its excellent diffusivity, H_2_ easily crosses biological membranes to reach the inside of mitochondria and protect cells from cellular damage caused by ·OH (41, 42). In our recent reviews, we reported that the protective effects of H_2_ on mitochondria may lead to preventive and therapeutic effects for chronic inflammatory diseases (44, 45).

Previous studies reported that drinking H_2_ water or inhaling H_2_ gas was effective in acute or chronic experiments on animals and humans. The administration of H_2_ to mice, rats, and racehorses subjected to acute or chronic exercise stress was found to exert anti-fatigue effects (46–49). Similar findings were obtained in healthy subjects who drank hydrogen-rich water (HRW) or inhaled H_2_ gas before or after exercise (50–57). The efficacy of HRW in patients with ME/CFS was suggested by Morris et al. (58) and Lucas et al. in their reviews (59). However, to the best of our knowledge, the efficacy of HRW or H_2_ gas inhalation remains unknown in patients with ME/CFS. Therefore, we herein reviewed the literature on the effects of H_2_ for acute or chronic fatigue and discussed the possible efficacy of H_2_ for ME/CFS.

## Criteria, Pathogenesis, and Etiology of ME/CFS

Since there is no specific diagnostic test for ME/CFS, a patient is initially examined for several other possible clinical diagnoses. If all are ruled out, the patient is then diagnosed according to the ME/CFS criteria. The main ME/CFS criteria used are the 1994 Fukuda Criteria (FC) (2), the 2003 Canadian Consensus Criteria (CCC) (60), the 2011 International Consensus Criteria (ICC) (1), and the 2015 Institute of Medicine Criteria (IOMC) (61). Of these, FC continues to be the most frequently used, although some argue that they are too broad in scope and overlap with other conditions to make an appropriate diagnosis; CCC, ICC, and IOMC include more ME/CFS-specific symptoms, such as post-exertional malaise. A major symptom common to all four criteria is the presence of persistent fatigue that does not improve with rest.

As described in the previous chapter, recent studies that conducted analyses of neuroimages and blood markers as well as energy metabolism and mitochondria revealed the presence of a number of objective biological abnormalities in ME/CFS (8). ME/CFS may be triggered by the activation of the immune system inside and outside the brain, resulting in the release of inflammatory cytokines (62, 63). These findings suggest that ME/CFS is associated with abnormalities related to the central and autonomic nervous systems, abnormalities in systemic energy metabolism, abnormalities in the immune system, and the involvement of oxidative and nitrosative stress (3). Systemic energy metabolism abnormalities have been explained by alterations in the structure and functions of mitochondria in the muscles and leukocytes of patients with ME/CFS, suggesting that mitochondrial dysfunction is involved in energy metabolism abnormalities in this disease (5–18). Recent studies reported that some of the “sequelae” of patients affected by coronavirus disease 2019 (COVID-19), which is raging worldwide, include ME/CFS-like diseases (64, 65).

ME/CFS is difficult to diagnose in general practice, and it may take many years before a diagnosis is confirmed. Furthermore, since there is no effective treatment, many patients are forced to stay at home and subsequently deteriorate. A double-blind, randomized, controlled trial on the antibody drug rituximab was recently conducted on patients with ME/CFS; however, its efficacy was not confirmed (66). Patients with ME/CFS may have malfunctioning mitochondria and metabolic pathways, resulting in various deficiencies, such as in the metabolism of fatty acids and amino acids, as well as inefficient ATP synthesis (67). Therefore, the use of supplements with protective effects against mitochondrial dysfunction has been attempted as part of treatment. The efficacies of supplements, such as nicotinamide adenine dinucleotide hydrogen (NADH), coenzyme Q10 (CoQ10), and acetyl L-carnitine (ALC), have been investigated (68–70). Although these substances exhibited some efficacy, their effects were limited (68–70). Therefore, the development of new therapeutic substances and treatments that are curative rather than symptomatic is desired.

## Effects of H_2_ on Mitochondrial Dysfunction

In mitochondria, the electron transfer system generates an electrochemical potential on the inner membrane side, and this electrochemical energy is converted into chemical energy for ATP. Although the mitochondrial inner membrane is a good insulator, electrons leak out at a certain frequency. Leaked electrons react with oxygen in the mitochondria to produce superoxide anions (${\text{O}}_{2}^{-}$) (41, 42, 71). ${\text{O}}_{2}^{-}$ is also produced at a certain frequency in the citric acid circuit in the mitochondria by α-ketoglutarate dehydrogenase. The ROS scavenging system is well developed *in vivo*, and ${\text{O}}_{2}^{-}$ is converted to hydrogen peroxide (H_2_O_2_) by superoxide dismutase (SOD), which is further converted to water by glutathione peroxidase or catalase (41, 42, 71). ${\text{O}}_{2}^{-}$ reduces transition metal ions, such as Fe^3+^ and Cu^2+^, which react with H_2_O_2_ to produce ·OH (Fenton reaction) (41, 42, 71). ·OH is produced by the reaction of ${\text{O}}_{2}^{-}$ and H_2_O_2_ catalyzed by transition metal ions (Heber-Weiss reaction). It is the most potent oxidizing ROS and indiscriminately reacts with nucleic acids, lipids, and proteins. Since H_2_ is a substance with excellent permeability to mitochondria, it may react with ·OH produced in mitochondria and convert it to water for detoxification (·OH + H_2_ → H· + H_2_O) (41–44, 71).

Oxidative stress in mitochondria induces chronic inflammation, which may contribute to ME/CFS, including acute and chronic fatigue. Inflammation is induced by the release of inflammatory cytokines, such as interleukin (IL)-1β and IL-18 by macrophages and dendritic cells in direct response to inflammatory triggering stimuli (44, 72). The production of these cytokines is transient in nature; however, when they are continuously produced due to some disturbance, acute inflammation is delayed, and chronic inflammation is triggered. Inflammasomes, an intracellular protein complex, play an important role in the production of IL-1β and IL-18 (44, 72–75). Among them, nucleotide-binding and oligomerization domain-like receptor family pyrin domain-containing 3 (NLRP3) inflammasomes play an important role in the production of IL-1β and IL-18 (44, 72–75). They are activated not only by pathogen-associated molecular patterns from silica, asbestos, and low osmolarity, but also by damage-associated molecular patterns as diverse stimuli from extrinsic factors, such as silica, asbestos, and low osmolarity to intrinsic factors, including ATP and urate crystals (44, 72–75).

Recent studies demonstrated that mitochondria-derived ROS significantly contributed to the activation of NLRP3 inflammasomes. ROS produced by poorly functioning mitochondria (mtROS) oxidize mitochondrial DNA (mtDNA), which then binds directly to NLRP3 to promote the formation of inflammasomes (44, 76). Caspase-1, which is activated in inflammasomes, processes the precursor forms of IL-1β and IL-18 into their mature forms, which are released into the extracellular space and induce inflammation (44). On the other hand, the activation of NLRP3 inflammasomes requires a preceding “priming” stimulus, typically lipopolysaccharide, which induces the expression of genes encoding the precursors IL-1β and NLRP3 via its receptor, toll-like receptor 4 (44, 77, 78). In our recent review, we demonstrated that among mtROS, ·OH may mainly promote the oxidation of mtDNA (44). As a mechanism for the amelioration of chronic inflammation by H_2_, we showed that the scavenging of ·OH in mitochondria may be involved in the suppression of the cascade from NLRP3 inflammasome activation to the release of inflammatory cytokines (44).

## Anti-Fatigue Effects of H_2_ and Underlying Mechanisms

**Figure 1:**
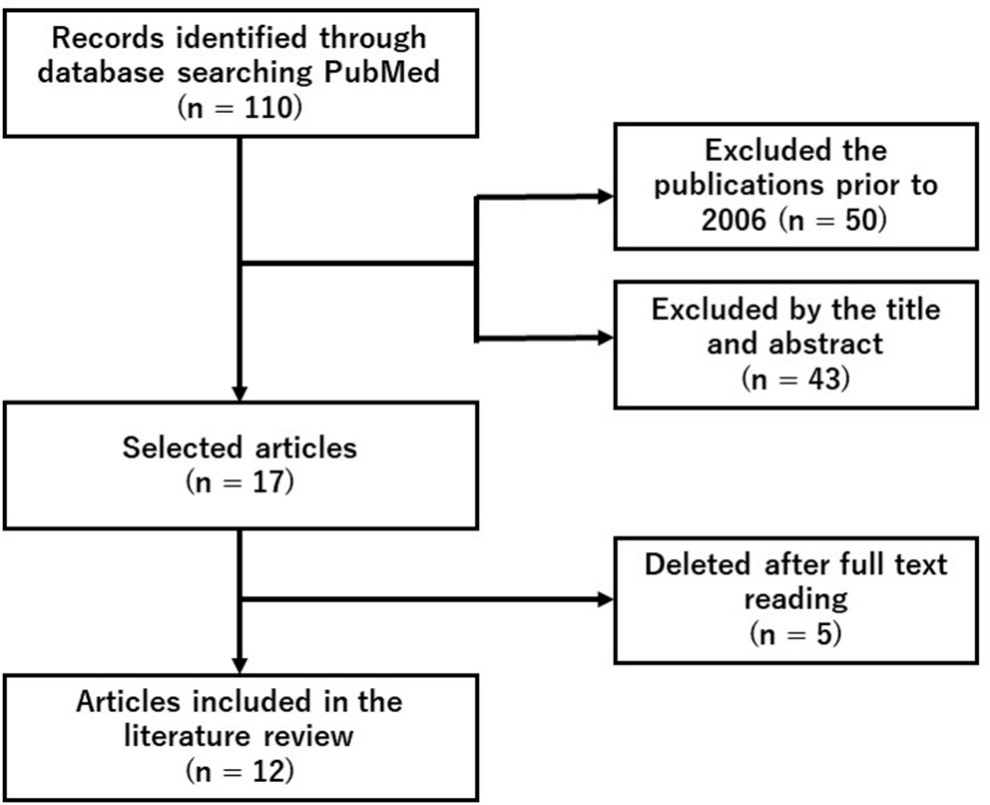
Flowchart representing literature's search and selection steps.

We did a systematic search of PubMed using the search terms (“hydrogen” and “fatigue” and “exercise”) on November 24, 2021. Since this search detected 12 original articles, we conducted a following literature review (Figure 1 and Table 1).

**Table 1:** Summary of anti-fatigue effects of molecular hydrogen (H_2_) in animal models and human clinical trials.

**Species**	**Type of H_**2**_**	**Effects of H_**2**_**	**Changes in biomarkers**	**References**
Mice	HRW	Anti-fatigue Anti-oxidation Anti-inflammation	Swimming endurance capacity↑, Blood glucose and lactate↓, Serum BUN↓, Liver glycogen↑, Serum LDH↑, Serum NO↓, Serum and liver GPx↑, Serum TNF-α, IL-6, IL-17, and liver IL-1β↓	(46)
Rats	H_2_ gas	Anti-oxidation Anti-inflammation Anti-stress	Plasma TNF-α and IL-6↓, Plasma TBARS↓, Plasma SOD↑, CREB in skeletal muscle↓	(47)
Horses	H_2_-saline	Anti-oxidation	Serum 8-OHdG↓	(48)
	HRW	Anti-oxidation	Serum d-ROM ↘, Serum BAP/d-ROM↑	(49)
Humans	HRW	Anti-fatigue	Blood lactate↓, Peak torque↑	(50)
	HRW	Anti-fatigue	Psychometric fatigue↓, Endurance↑, Fatigue judged by Borg's scale↓	(51)
	HRW	Anti-fatigue	Blood lactate↓, Ventilatory equivalent for oxygen and RPE↓	(52)
	H_2_ gas	Anti-fatigue Anti-oxidation	Urinary 8-OHdG excretion rate↓, Countermovement jump height↑	(53)
	HRW	Anti-oxidation	Serum BAP/d-ROM↑	(54)
	HRW	Anti-fatigue	Peak oxygen uptake↑, Peak load ↗	(55)
	HRW	Anti-fatigue	Peak power↑, Mean power↑, Fatigue index↓	(56)
	HRW	Anti-fatigue	PPO↑, ΔPPO↑	(57)

*H_2_, molecular hydrogen; HRW, hydrogen-rich water; BUN, blood urea nitrogen; LDH, lactate dehydrogenase; NO, nitric oxide; GPx, glutathione peroxidase; TNF-α, tumor necrosis factor-α; IL, interleukin; TBARS, thiobarbituric acid reactive species; SOD, superoxide dismutase; CREB, cAMP-responsive element-binding protein; 8-OHdG, 8-hydroxydeoxyguanosine; d-ROM, diacron-reactive oxygen metabolites; BAP, biological antioxidant potential; RPE, ratings of perceived exertion; PPO, peak power output; Ref., reference; ↓, significant decrease; ↑, significant increase; ↘, slight decrease; ↗, slight increase*.

H_2_ exerted anti-fatigue effects in mice, rats, and racehorses subjected to acute or chronic exercise loading (46–49). Similarly, the anti-fatigue effects of H_2_ on healthy subjects who performed acute or chronic exercise have been investigated (50–57). In this chapter, we provide an overview of the specific anti-fatigue effects of H_2_ in animal models and human clinical trials, and also discuss the underlying mechanisms (Table 1).

### Effects of H_2_ in Animal Models

Ara et al. investigated the effects of HRW (1.0–1.2 ppm) on fatigue in mice subjected to daily stress-induced swimming for 4 weeks (46). The findings obtained showed higher swimming endurance in the HRW group than in the placebo water (PW) group (46). In addition, blood glucose, lactate, and serum BUN levels were significantly lower, while liver glycogen and serum LDH levels were significantly higher in the HRW group than in the PW group. Moreover, the HRW group showed decreased nitric oxide (NO) and increased glutathione peroxidase (GPx) levels as well as decreased serum tumor necrosis factor-α (TNF-α), IL-6, and IL-17 levels and liver IL-1β levels (46). These findings suggest that HRW exerted its anti-fatigue effects through metabolic regulation, the redox balance, and the inhibition of inflammation.

Nogueira et al. examined the anti-fatigue effects of H_2_ gas (2%) on rats subjected to acute exercise loading on a treadmill (47). Immediately and 3 h after exercise, rats were euthanized and inflammatory markers in plasma were measured. Skeletal muscle was also collected to examine the phosphorylation status of intracellular signaling proteins. The findings obtained showed that H_2_ gas suppressed exercise-induced elevations in inflammatory cytokines (TNF-α and IL-6) and thiobarbituric acid reactive species (TBARS), as well as further increases in SOD (47). H_2_ gas suppressed the phosphorylation of skeletal muscle cAMP-responsive element-binding protein (CREB) (47). Nogueira et al. suggested that H_2_ plays an important role in the attenuation of exercise-induced inflammation, oxidative stress, and cellular stress (47).

Yamazaki et al. examined the effects of intravenously administered H_2_-containing saline solution (H_2_-saline, 0.6 ppm) on oxidative stress in racehorses prior to their participation in a high-intensity simulation race (48). No significant differences were observed in the biological antioxidant potential (BAP) and diacron-reactive oxygen metabolites (d-ROM), between horses administered H_2_-saline and a placebo. However, H_2_-saline significantly inhibited 8-hydroxydeoxyguanosine (8-OHdG) at all-time points: immediately after the race, 3 h later, and 24 h later (48). These findings indicate that H_2_-saline significantly inhibited oxidative stress induced after exhausting races (48).

Tsubone et al. also investigated the effects of HRW (1 ppm) on oxidative stress and the antioxidant capacity in response to treadmill exercise in racehorses (49). HRW and PW were administered orally 30 min before treadmill exercise, and blood samples were collected. In comparisons with PW, HRW slightly reduced d-ROM from the pre-exercise value and significantly increased BAP/d-ROM immediately before exercise, immediately after exercise, and 30 min after exercise (49). Tsubone et al. demonstrated that HRW attenuated oxidative stress induced by treadmill exercise (49). However, we question their results because the difference between the placebo and HRW groups is extremely small.

### Effects of H_2_ in Human Clinical Trials

Intense exercise for a short period of time may induce oxidative stress, which may, in turn, contribute to the development of overtraining symptoms, such as increased fatigue, resulting in muscle microdamage and inflammation. Aoki et al. investigated the effects of HRW on oxidative stress and muscle fatigue during acute exercise (50). Athletes ingested HRW (2.0 ppm) or PW, followed by exercise loading with a cycle ergometer and maximal isometric knee extension. In comparisons with PW, HRW did not induce significant changes in d-ROM, BAP, and creatine kinase after exercise (50). However, it significantly suppressed increases in blood lactate levels (50). It also inhibited the initial decrease in peak torque observed with PW (50). These findings suggest that HRW may reduce blood lactate levels and ameliorate exercise-induced muscle dysfunction.

Mikami et al. examined the effects of HRW on psychological fatigue and endurance in response to exercise loading. In experiment 1, all healthy untrained subjects ingested HRW (0.8 ppm) or PW 30 min before light exercise on a cycle ergometer (51). Psychological fatigue was significantly lower in the HRW group than in the PW group (51). In Experiment 2, trained participants were subjected to moderate exercise with a cycle ergometer 10 min after the ingestion of HRW (1.0 ppm) using the same method as that in Experiment 1. Based on maximal oxygen consumption and the Borg's scale, significant improvements were observed in endurance and fatigue in the HRW group (51). Therefore, Mikami et al. suggested that HRW may contribute to recovery and better endurance (51). Since the effects of HRW in the present study were clinically negligible, the anti-fatigue and endurance-enhancing effects of HRW have been questioned, although this has been refuted by the authors (79, 80).

HRW may be useful for recovery and enhancing performance. Botek et al. evaluated the physiological and perceptual efficacies of HRW in a protocol in which HRW (0.5 ppm) was administered to healthy volunteers within 30 min before exercise and exercise intensity was progressively increased (52). Cardiopulmonary function, lactate levels, and the rating of perceived exertion (RPE) were examined during the last minute of each exercise step. The findings obtained revealed that blood lactate levels, the ventilatory equivalent of oxygen, and RPE were significantly lower in the HRW group than in the PW group (52). These findings suggest that HRW reduced blood lactate levels at higher exercise intensities and enhanced the sense of effort and ventilatory efficiency of exercise (52).

Shibayama et al. examined the effects of acute H_2_ gas inhalation on subsequent oxidative stress, muscle damage, and exercise performance during recovery after intense exercise (53). Volunteers performed oxidative stress-inducing exercise consisting of treadmill running and squat jumps for 30 min, and then inhaled H_2_ gas (68%) or placebo gas for 60 min (recovery period) (53). In comparisons with the placebo, H_2_ gas significantly reduced the urinary 8-OHdG excretion rate and increased the height of countermovement jumps (53). These findings suggest that H_2_ gas enhanced exercise performance by reducing systemic oxidative damage.

Continuous sprinting exercise may disrupt the redox balance in muscles, causing systemic oxidative stress and muscle damage. Dobashi et al. investigated the effects of HRW on oxidative stress and muscle fatigue in healthy subjects subjected to 3 days of continuous exercise loading (54). PW and HRW (5 ppm) were consumed before and after each exercise session. Blood samples were collected 7 h before the first exercise session (day 1) and 16 h after each exercise session. The findings obtained showed that the relative change from baseline in BAP/d-ROM, an index of antioxidant capacity, decreased over time in the PW group (54). However, in the HRW group, the decrease in BAP/d-ROM observed in the PW group was significantly suppressed (54). These findings suggest that HRW contributed to maintaining the redox status during continuous intense exercise and prevented the accumulation of muscle fatigue.

Although animal studies reported that H_2_ enhanced mitochondrial metabolism, the effects of H_2_ on aerobic capacity during exercise in humans remain unclear. Hori et al. investigated whether the continuous intake of HRW (5.9 ppm) for 2 weeks by healthy subjects increased aerobic capacity during gradual cycling exercise (55). In comparisons with PW, HRW significantly increased peak oxygen uptake and slightly increased the peak load (55). Hori et al. suggested that HRW enhanced aerobic exercise performance and physical health. They also indicated that the enhancement in aerobic capacity during exercise with HRW was due to increased mitochondrial energy production (55).

Timón et al. examined the effects of the weekly intake of HRW on aerobic and anaerobic exercise performance in both trained and untrained humans (56). Two experimental groups, trained cyclists and untrained subjects, ingested PW and nanobubbled HRW (1.9 ppm). The findings obtained showed enhanced performance in trained cyclists only in the anaerobic test, with increased peak and average power as well as a reduced fatigue index (56). These findings indicate that the ergogenic effects of HRW are dependent on the state of training. Furthermore, HRW appeared to effectively enhance the anaerobic performance of trained cyclists (56).

Da Ponte *et al*. investigated the effects of 2 weeks of HRW ingestion on repetitive sprint performance and the acid-base status during prolonged intermittent cycling exercise (57). Trained male cyclists were given PW or HRW (0.45 ppm) daily and tested at baseline and after each 2-week period of treatment. The findings obtained showed that the absolute value of peak power output (PPO) was significantly reduced in the PW group on the 8th and 9th out of 10 sprints, and the relative value of ΔPPO was significantly reduced on the 6th, 8th, and 9th sprints; however, these decreases were significantly attenuated in the HRW group (57). These findings indicate that HRW contributed to the maintenance of PPO in repeated sprints to exhaustion (57).

### Possible Mechanisms Underlying Fatigue-Ameliorating Effects

H_2_ has been reported to exert anti-fatigue effects in experimental animals and healthy subjects by increasing exercise capacity (46, 50–53, 55–57), reducing fatigue indices (51, 56), and inhibiting increases in blood lactate levels due to muscle fatigue (46, 50, 52). H_2_ was also found to inhibit increases in cAMP-responsive element-binding protein (CREB), a marker of the phosphorylation of intracellular signaling proteins in skeletal muscle associated with exercise (47). On the other hand, d-ROM, 8-OHdG, and TBARS have been used as oxidative stress markers, and BAP, GPx, BAP/d-ROM, and SOD as antioxidant markers, and the effects of H_2_ on these markers has also been evaluated. H_2_ reduced TBARS (47), d-ROM (49), and 8-OHdG (48, 53), and increased GPx (46), SOD (47), and BAP/d-ROM (49, 54). Furthermore, H_2_ decreased inflammatory markers, such NO, TNF-α, IL-1β, IL-6, and IL-17 (46, 47). These findings suggest that enhancements in athletic performance and anti-fatigue effects by H_2_ are due to its antioxidant and anti-inflammatory effects (Table 1).

Recent studies demonstrated that H_2_ exhibited antioxidant and other biological activities through the regulation of various types of gene expression, in addition to its scavenging effects on ROS, which are often produced by mitochondria (47, 51, 64–66). Nogueira et al. (47) suggested that the mechanism by which H_2_ inhibits oxidation, inflammation, and cellular stress in rats subjected to acute exercise stress involved the regulation of gene expression. Furthermore, Sobue et al. (81) proposed that H_2_ activates mitochondrial unfolded protein responses and exerts biological effects via epigenetic histone modifications and changes in gene expression. Hori et al. (56) indicated that the continuous intake of HRW increased mitochondrial energy production via the expression of these genes and proteins, resulting in an increased peak oxygen consumption during progressive exercise. On the other hand, Mizuno et al. reported the ameliorating effects of HRW on mood, anxiety, and autonomic nerve function in daily life (82), and Hu et al. demonstrated that electrolyzed H_2_ water attenuated chronic stress due to its antioxidant and anti-inflammatory effects (83). We also demonstrated in a recent review that the radio-protective and anti-tumor effects of H_2_ may involve not only its direct scavenging effects on ·OH, but also its antioxidant and anti-inflammatory effects through the regulation of gene expression as indirect effects (31, 71). Therefore, although further genetic studies are needed, enhancements in athletic performance by H_2_ and the anti-fatigue effects of H_2_ involve not only direct scavenging effects on mitochondria-generated ROS, but also antioxidant and anti-inflammatory effects through the regulation of gene expression as indirect effects.

## Dose H_2_ Improve Mitochondrial Disorders in ME/CFS?

Abnormalities in the structure and functions of mitochondria have been detected in patients with ME/CFS (5–25). Studies on the structure of mitochondria in the muscles and leukocytes of patients with ME/CFS revealed the enrichment of mitochondrial cristae (5), polymorphisms in mitochondrial DNA (6), and relationships between specific haplotypes in mitochondrial DNA and specific symptoms (7). Regarding mitochondrial dysfunction, metabolomics studies suggested abnormalities in energy production pathways from monosaccharides, fatty acids, and amino acids (5–11). Previous studies on patients with ME/CFS reported increases (5) and decreases in ATP synthesis (17) in leukocytes, while elevated lactate levels in cerebrospinal fluid indicated impaired oxidative phosphorylation and, as a result, increased anaerobic metabolism (84). Furthermore, decreases in NADH and CoQ10 have been observed in patients with ME/CFS (85).

**Figure 2:**
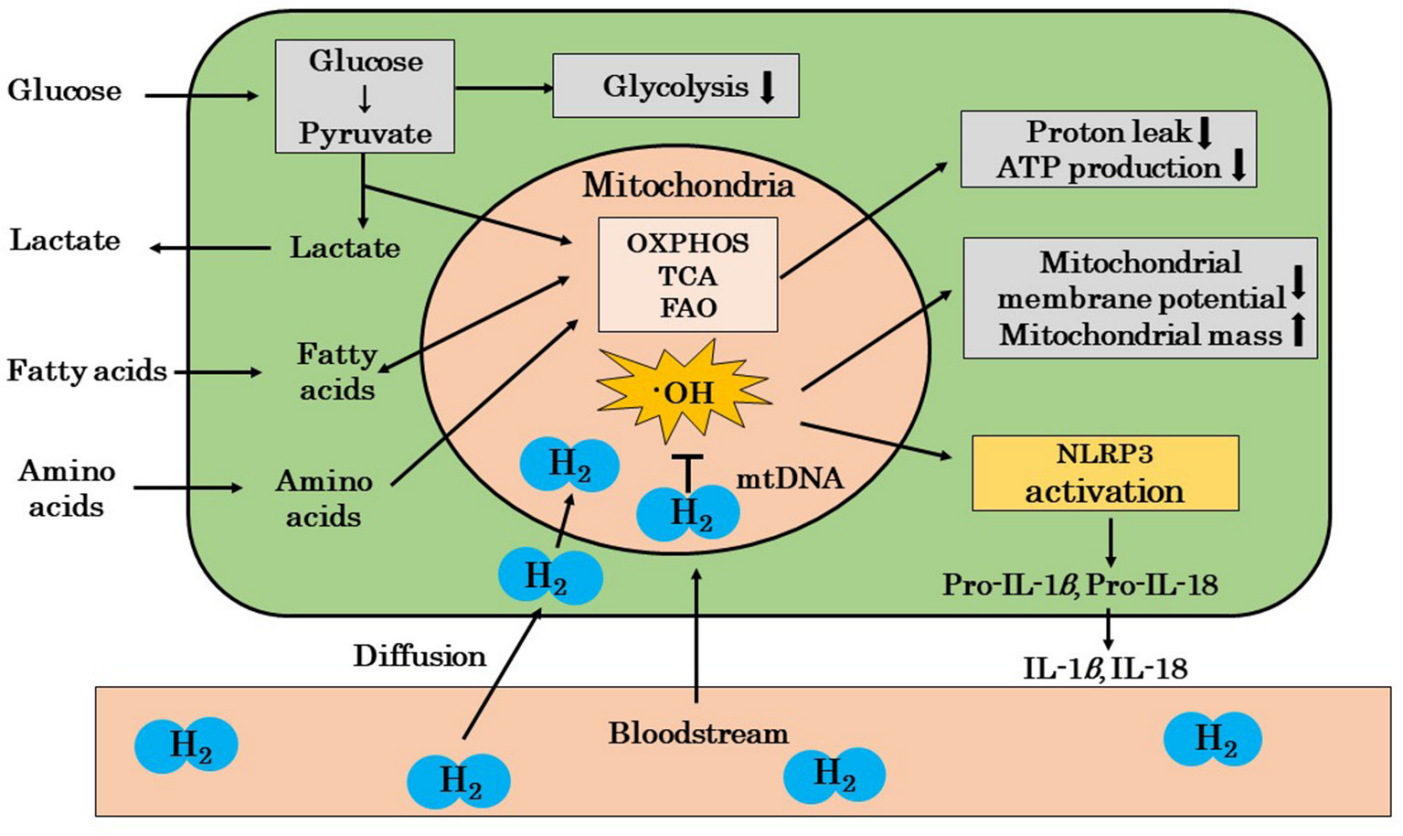
A possible mechanism by which H_2_ ameliorates mitochondrial dysfunction in ME/CFS patients. The mitochondria of ME/CFS patients show a reduced glycolytic capacity and abnormal metabolism. These mitochondria show decreased proton leakage, ATP production, and mitochondrial membrane potential and an increased mitochondrial mass. H_2_ ameliorates mitochondrial dysfunction by selectively scavenging ·OH, which is the cause of mitochondrial damage, and blocks the cascade from NLRP3 activation to the release of inflammatory cytokines, such as IL-1β and IL-18. H_2_, molecular hydrogen; ME/CFS, myalgic encephalomyelitis/chronic fatigue syndrome; ·OH, hydroxyl radicals; OXPHOS, oxidative phosphorylation; TCA, tricarboxylic acid cycle; FAO, fatty acid oxidation; mtDNA, mitochondrial DNA; IL, interleukin; NLPR3, nucleotide-binding and oligomerization domain-like receptor family pyrin domain-containing 3 (inflammasome); ↓, decrease; ↑, increase.

Mandarano et al. reported that the mitochondrial membrane potential was reduced in the CD8^+^ T cells of patients with ME/CFS both at rest and during activation (86). Hornig also showed that resting glycolysis was impaired in the CD4^+^ and CD8^+^ T cells of patients with ME/CFS, and CD8^+^ T cells showed impaired activation-related metabolic remodeling and a reduced mitochondrial membrane potential (87). Furthermore, Hornig suggested that mitochondrial ROS induced the activation of NLRP3 in ME/CFS patients, and that the release of IL-1β and IL-18 may induce inflammation (87). We showed in a recent review that ·OH may mainly promote the oxidation of mitochondrial DNA (44). We also reported that the amelioration of chronic inflammation by H_2_ may be explained by the scavenging of ·OH in mitochondria, which inhibits the cascade from the activation of NLRP3 inflammasomes to the release of IL-1β and IL-18 (44). These findings suggest that H_2_ protects against mitochondrial dysfunction by scavenging mitochondria-produced ·OH in patients with ME/CFS (Figure 2).

## Prospects of H_2_ as a Therapeutic Substance for ME/CFS

The development of therapies and drugs for ME/CFS has been vigorously pursued. Chinese herbal medicine has been applied as a treatment for decreased immunity, such as the decreased activation of natural killer cells, and vitamin C, NADH, and CoQ10 have been administered as antioxidant-enhancing drugs for increased oxidative stress and decreased antioxidant capacity (68–70). Furthermore, non-steroidal anti-inflammatory drugs have been administered to patients with severe myalgia, arthralgia, and headache, and vaccinia virus-inoculated rabbit inflammatory skin extracts have been applied to the treatment of patients with concomitant neuropathic pain (88). However, all of these therapies are symptomatic and not curative treatments that focus on the etiology of ME/CFS.

In this study, we reported that H_2_ was effective against fatigue induced by acute and chronic exercise stress in animals and healthy subjects by a literature review (46–57). In addition to the direct scavenging of mitochondria-generated ROS, H_2_ may also exert indirect antioxidant and anti-inflammatory effects through the regulation of various types of gene expression as the mechanisms of anti-fatigue effects (31, 47, 51). The findings of a literature review also suggested that the protection against mitochondrial dysfunction may be partly involved in the amelioration of H_2_ on the acute and chronic fatigue in animals and healthy subjects. Since mitochondrial dysfunction plays a major role in abnormal energy metabolism in ME/CFS, our literature review suggested that H_2_ gas may be an effective medical gas for the treatment of ME/CFS (58, 59, 62, 63). More than 1,300 studies on H_2_ have been reported to date, including ~100 clinical trials, and research on the medical use of H_2_ is being conducted worldwide (43). Since H_2_ is produced by intestinal bacteria (89) and is recognized as a food additive in Japan, the U.S., and European Union (EU), and H_2_ gas has been applied to the treatment of treat caisson disease (90), there are no safety issues associated with H_2_. Therefore, further clinical studies to evaluate the efficacy of H_2_ gas as a therapeutic substance for ME/CFS are needed.

Although the anti-fatigue effects of H_2_ have been reported in animal studies, these experiments were conducted using HRW or H_2_-saline. These effects have been reported in clinical trials; however, only Nogueira et al. (47) and Shibayama et al. (53) used H_2_ gas, while the others used HRW or H_2_-saline. Moreover, Morris et al. (58) and Lucas et al. (59) indicated the efficacy of HRW in ME/CFS patients in their reviews. These studies showed the potential anti-fatigue effects of HRW or the possible efficacy of HRW in ME/CFS patients, but not the efficacy of H_2_ gas inhalation. Liu et al. (91) examined the effects of the oral administration of HRW and H_2_ gas inhalation in rats and measured the blood and tissue concentrations of H_2_ over time. The findings obtained showed that the maximum values of blood and tissue concentrations were higher with the oral administration of HRW than with H_2_ gas inhalation, while the area under the curve of these values markedly increased with H_2_ gas inhalation in a time-dependent manner. Therefore, the efficacy of H_2_ in patients with ME/CFS appears to be greater following H_2_ gas inhalation than with HRW.

## Ameliorative Effect of H_2_ on COVID-19 “Sequelae”

COVID-19 is caused by Severe Acute Respiratory Syndrome Coronavirus 2 (SARS-CoV-2), a viral infection that induces a number of respiratory, digestive, and vascular symptoms. Symptoms in the acute phase generally subside within 2–3 weeks. However, some patients with COVID-19 have a prolonged recovery period and may continue to have “sequelae” for months after the initial infection. A number of chronic symptoms have been reported as these “sequelae,” including fatigue, dyspnea, myalgia, exercise intolerance, sleep disturbances, poor concentration, anxiety, fever, headache, and malaise (64, 65). These symptoms have been described as “long COVID” or “post COVID” and are similar to those observed in ME/CFS (64, 65). However, despite these similarities, there is currently no evidence to show that COVID-19 is a trigger for ME/CFS (19).

Guan et al. (37) examined the effects of a H_2_/O_2_ mixed gas (67% H_2_, 33% O_2_) on patients with COVID-19 by an open-label multicenter clinical trial and showed that the improvements in disease severity, dyspnea, cough, chest distress, chest pain, and oxygen saturation were significantly greater in H_2_/O_2_ treatment group (44 patients) than those in control group (46 patients). In addition, Botek et al. recently reported the results of a randomized, single-blind, placebo-controlled study of the effects of 14 days of H_2_ gas inhalation (2 × 60 min/day) on the physical and respiratory status of 50 acute “post COVID-19” patients. Compared to the placebo gas, H_2_ gas inhalation significantly improved physical and respiratory function on gait and pulmonary function tests, suggesting that H_2_ gas may improve the symptoms of acute “post COVID-19” patients (92). These results indicate that inhalation of H_2_ gas may have a therapeutic effect not only on patients with COVID-19 but also on those with “post COVID-19.”

Although further research is needed in the future, if we assume that “long COVID” or post COVID” develops through a similar mechanism to ME/CFS, H_2_ gas inhalation may be useful for the treatment of ME/CFS including “long COVID” or “post COVID.”

## Conclusion

Since H_2_ ameliorates mitochondrial dysfunction (43), we herein reviewed the literature for the anti-fatigue effects of H_2_ in animal studies and human clinical trials. The findings of the literature review suggested that H_2_ exerts anti-fatigue effects, and that these effects may involve not only the direct scavenging of mitochondria-generated ROS by H_2_, but also its antioxidant and anti-inflammatory effects through the regulation of gene expression (31, 46, 50). Since mitochondrial dysfunction is also involved in the etiology of ME/CFS (5–25), the literature review also suggested that the anti-fatigue effects of H_2_ in animal and human clinical studies indicate a possible ameliorative effect of H_2_ on ME/CFS (57, 58, 62, 63). Since “long COVID” or “post COVID,” the “sequelae” of COVID-19, may be similar to ME/CFS (64, 65), there is an urgent need to develop precise therapies and substances for ME/CFS. H_2_ gas may be an effective medical gas for the treatment of ME/CFS.

## Author Contributions

S-iH performing study and design and drafting the manuscript. YI, BS, YT, and FS revising the manuscript for important intellectual content. All authors have read and agree to the published version of the manuscript.

## Conflict of Interest

S-iH, YI, BS, and FS are employed by MiZ Company Limited. The remaining author declares that the research was conducted in the absence of any commercial or financial relationships that could be construed as a potential conflict of interest.

## Publisher's Note

All claims expressed in this article are solely those of the authors and do not necessarily represent those of their affiliated organizations, or those of the publisher, the editors and the reviewers. Any product that may be evaluated in this article, or claim that may be made by its manufacturer, is not guaranteed or endorsed by the publisher.
